# Novel Proteome and N-Glycoproteome of the Thermophilic Fungus *Chaetomium thermophilum* in Response to High Temperature

**DOI:** 10.3389/fmicb.2021.644984

**Published:** 2021-06-07

**Authors:** Jinpeng Gao, Qingchao Li, Duochuan Li

**Affiliations:** Department of Mycology, Shandong Agricultural University, Taian, China

**Keywords:** proteome, N-glycoproteome, *Chaetomium thermophilum*, thermophily, glycosylation

## Abstract

Thermophilic fungi are eukaryotic species that grow at high temperatures, but little is known about the underlying basis of thermophily at cell and molecular levels. Here the proteome and N-glycoproteome of *Chaetomium thermophilum* at varying culture temperatures (30, 50, and 55°C) were studied using hydrophilic interaction liquid chromatography enrichment and high-resolution liquid chromatography–tandem mass spectroscopy analysis. With respect to the proteome, the numbers of differentially expressed proteins were 1,274, 1,374, and 1,063 in T50/T30, T55/T30, and T55/T50, respectively. The upregulated proteins were involved in biological processes, such as protein folding and carbohydrate metabolism. Most downregulated proteins were involved in molecular functions, including structural constituents of the ribosome and other protein complexes. For the N-glycoproteome, the numbers of differentially expressed N-glycoproteins were 160, 176, and 128 in T50/T30, T55/T30, and T55/T50, respectively. The differential glycoproteins were mainly involved in various types of N-glycan biosynthesis, mRNA surveillance pathway, and protein processing in the endoplasmic reticulum. These results indicated that an efficient protein homeostasis pathway plays an essential role in the thermophily of *C*. *thermophilum*, and N-glycosylation is involved by affecting related proteins. This is the novel study to reveal thermophilic fungi’s physiological response to high-temperature adaptation using omics analysis, facilitating the exploration of the thermophily mechanism of thermophilic fungi.

## Introduction

Glycosylation, the attachment of glycans to proteins, is a posttranslational modification to produce significant structural changes to proteins (Ge et al., 2018). Protein glycosylation is common in all kinds of life (bacteria, archaea, and eukaryotes), demonstrating multiple cell functions, such as protein folding, signal transduction, stability, targeting, cell–cell interactions, and host immune response (Mitra et al., 2006; Vigerust and Shepherd, 2007; Calo et al., 2010; Nothaft and Szymanski, 2010; Eichler, 2020). Glycosylation is considered the most complicated posttranslational modification due to the multiple enzymatic steps (Eichler, 2020). Glycans are encoded in a complex dynamic network containing hundreds of genes, which form the enzymes for glycan synthesis. Molecular events, such as transferring sugars from one substrate to another, linking monosaccharides, and trimming sugars from glycan structures, are involved in glycosylation. Glycosylation is non-templated, and cells employ a host of enzymes to add or remove sugars from one molecule to another to generate glycoproteins in a given cell (Eichler, 2020). Importantly, glycosylation enhances the proteome’s diversity, as almost any aspect of glycosylation is modifiable, such as glycosidic linkage and glycan composition, structure, and length (Zacchi and Schulz, 2016). N-linked glycans (N-glycans) are attached to an Asn residue in a defined protein sequence, Asn–X–Ser/Thr (X refers to an amino acid other than Pro) (Apweiler et al., 1999). N-linked glycans are derived from a core 14-sugar unit assembled in the cytoplasm and endoplasmic reticulum in eukaryotes. N-linked glycan’s core structure comprises 14 residues (three glucose residues, nine mannose residues, and two N-acetyl glucosamine residues) (Munro, 2009).

Generally, thermophilic fungi grow at a maximum temperature of 50°C and a minimum temperature of 20°C (Morgenstern et al., 2012). Owing to the potential value of thermostable enzymes in many biotechnological applications, thermophilic fungi have received significant attention. A lot of thermostable enzymes from thermophilic fungi have been purified, cloned, expressed, and characterized. Furthermore, the available crystal structures of thermophilic fungal enzymes provide more insights into their functions and stabilities (Maheshwari et al., 2000; Li et al., 2010, 2011; Haikarainen et al., 2014; Papageorgiou et al., 2017). Genomic sequencing, transcriptome data, and secreted proteins revealed several enzymes involved in biomass degradation in thermophilic fungi (Berka et al., 2011). Interestingly, recent phylogenetic analysis suggests that thermophily of thermophilic fungi may be gained independently by convergent evolution (Berka et al., 2011; Morgenstern et al., 2012; van den Brink et al., 2015). However, the molecular basis for fungal thermophily is still unclear, and whether protein glycosylation is involved in fungal thermophily is unknown. Although most thermophiles actually have high GC content, the genomes of two thermophilic fungi show a slightly lower genome-level GC content than those of a mesophilic fungus, suggesting that high GC content is not essential for fungal thermophily (Berka et al., 2011; Muggia et al., 2020). Comparing the proteomes from archaea and bacteria, a seven-amino acid motif IVYWREL is positively correlated with high growing temperature in thermophilic prokaryotes, but the fungal genome analysis found that the motif is not positively correlated with high growing temperature in thermophilic and mesophilic fungi (Berka et al., 2011). On the basis of a further thermophilic and mesophilic fungal genome comparison of heat shock proteins (Hsps), chromatin structure and modification, membrane biosynthesis, oxidative stress, and cell wall metabolism, no differences can be interpreted by fungal thermophily (Berka et al., 2011).

High-temperature adaptation has attracted widespread attention. The latest review gave an overview and summary of fungal proteomics under temperature stress (Abu Bakar et al., 2020). The 2D protein gels of *Friedmanniomyces endolithicus* shows that the amount of protein decreases under high-temperature pressure, indicating a lack of heat shock response (Tesei et al., 2012). RNA-seq shows that *Exophiala dermatitidis* increases Golgi activity and protein transport at high temperature and increases lipid metabolism, the post-chaperonin tubulin folding pathway and cellular developmental processes at low temperature (Blasi et al., 2015). Under high temperature, *Aspergillus niger* proteins related to cellular signaling, carbohydrate metabolism, and cell wall organization are significantly upregulated based on iTRAQ proteomic analysis (Deng et al., 2020). *Aspergillus flavus* proteomic analysis shows that carbohydrate and energy metabolism, signal transduction, and protein metabolism are important responses to heat stress (Zou et al., 2018). Analysis of peptidases revealed an increase in amino acids Ala, Glu, Gly, Pro, Arg, and Val, suggesting that charged and hydrophobic residues can improve thermal stability by improving electrostatic interaction, hydrophobic interaction, and protein rigidity (Sokalingam et al., 2012; Wang et al., 2014; de Oliveira et al., 2018).

*Chaetomium thermophilum* grows at temperatures up to 55°C. This model thermophilic fungus holds promise for studying biochemical structural analyses of macromolecular complexes and biotechnological uses of thermostable eukaryotic proteins (Li et al., 2010; Amlacher et al., 2011; Kellner et al., 2016). Here, a great many differentially expressed proteins and N-glycoproteins of *C. thermophilum* grown at 30, 50, and 55°C were identified by proteomics and glycomics analysis. These results indicate that protein homeostasis pathways play a key role in the thermophily of *C. thermophilum*. In addition, N-glycosylation appears to be one of the processes that allow for the thermophily of *C. thermophilum*. This study may thereby provide insights into the relationship between protein biochemistry, including protein glycosylation, and adaptation to high temperature.

## Results

### *C. thermophilum* Growth and Cellular Proteins at 30, 50, and 55°C

**FIGURE 1:**
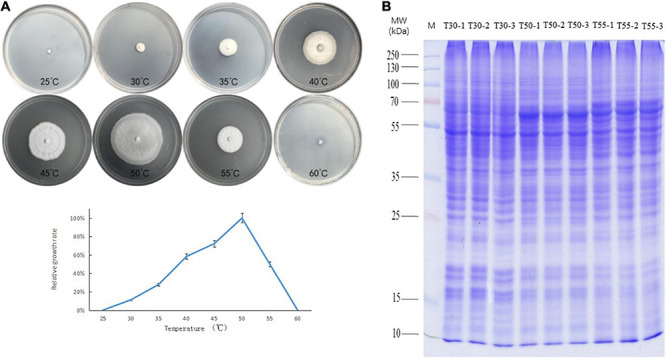
Growth of *C. thermophilum* and SDS-PAGE of cell proteins. **(A)** Growth of *C. thermophilum* at different temperatures, 25, 30, 35, 40, 45, 50, 55, and 60°C, after 3 days. **(B)** SDS-PAGE of cellular proteins from *C. thermophilum* mycelia growing at three different temperatures 30°C (T30), 50°C (T50), and 55°C (T55). Each sample was loaded in triplicate, as indicated by labels 1, 2, and 3; Lane M, molecular mass markers.

The growth of *C*. *thermophilum* after 3 days was compared at temperatures from 25 to 60°C at intervals of 5°C (Figure 1A). The minimum, optimum, and maximum growth temperatures of *C*. *thermophilum* were 30, 50, and 55°C, respectively. Therefore, *C*. *thermophilum* mycelia growing at 30, 50, and 55°C after 3 days were selected to analyze the proteome and N-glycoproteome. *C*. *thermophilum* mycelia were homogenized in 8 M urea and a buffer containing 1% Triton X-100 (Figure 1B).

### Overview of Proteome and N-Glycoproteome in *C. thermophilum*

**FIGURE 2:**
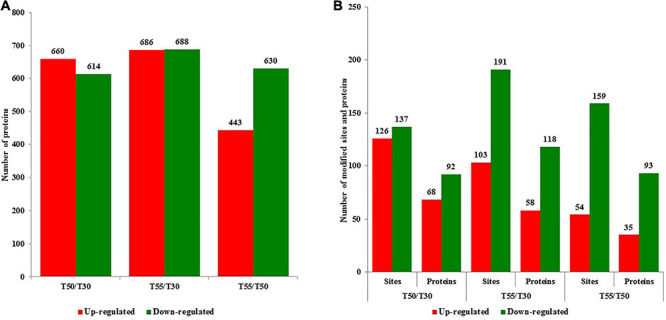
The number of upregulated and downregulated proteins and N-glycoproteins with fold change over 1.5-fold. **(A)** The number of upregulated and downregulated proteins in T50/T30, T55/T30, and T55/T50, respectively. **(B)** The number of upregulated and downregulated N-glycoproteins in T50/T30, T55/T30, and T55/T50, respectively.

To identify cell proteins and N-glycoproteins from *C*. *thermophilum* at different temperatures, the proteome and N-glycoproteome of *C*. *thermophilum* were analyzed successively. The proteome and N-glycoproteome have been submitted to the PRIDE database (accession ID: PXD023311)^1^. The identification of 4,302 proteins was performed in *C*. *thermophilum*, among which the quantification of 3,878 proteins was completed with TMT labeling (Supplementary Table 1). On the basis of the criteria of fold change ratio > 1.5 and *p* < 0.05, 1,274, 1,374, and 1,063 proteins were differentially expressed in T50/T30, T55/T30, and T55/T50, respectively (Figure 2A). Among differentially expressed proteins, there were 660, 680, and 443 upregulated proteins and 614, 688, and 630 downregulated proteins in T50/T30, T55/T30, and T55/T50, respectively. The identification of 570 N-glycosylation sites in 316 protein groups was performed, of which the quantification of 498 sites in 278 proteins was accurately completed (fold change ratio > 1.5 and *p* < 0.05; Supplementary Table 2). The number of upregulated N-glycoproteins was 68, 58, and 35 in T50/T30, T55/T30, and T55/T50, respectively (Figure 2B). The number of downregulated N-glycoproteins was 92, 191, and 93 in T50/T30, T55/T30, and T55/T50, respectively.

**FIGURE 3:**
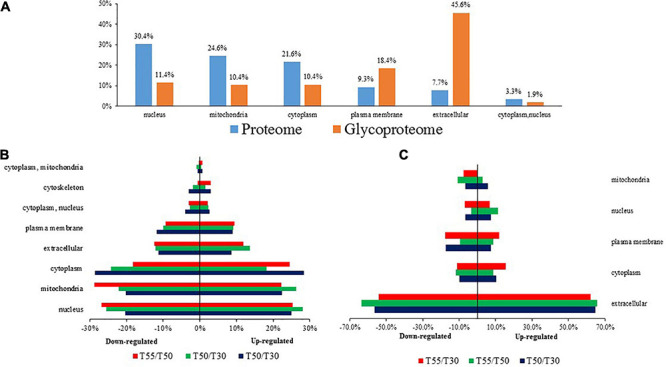
The subcellular localization of *C. thermophilum* proteome and N-glycoproteome data. **(A)** The distribution of proteins and N-glycoproteins in subcellular locations. **(B)** Subcellular locations of differentially expressed proteins in T50/T30, T55/T30, and T55/T50. **(C)** Subcellular locations of differentially expressed N-glycoproteins in T50/T30, T55/T30, and T55/T50.

Subcellular prediction was performed to confirm the subcellular localization of differentially expressed N-glycoproteins and proteins (Figure 3 and Supplementary Table 3). Most differentially expressed proteins were distributed in the nucleus (30.4%), mitochondria (24.6%), cytoplasm (21.6%), and extracellular region (7.7%). In contrast, most differentially expressed N-glycoproteins were distributed in the extracellular region (45.6%), plasma membrane (18.4%), nucleus (11.4%), mitochondria (10.4%), and cytoplasm (10.4%; Figure 3A). Differentially expressed proteins and N-glycoproteins were then separately analyzed, and the distribution in different locations was found (Figures 3B,C). Therefore, most differentially expressed proteins were distributed in the cytoplasm, nucleus, and mitochondria, and most differentially expressed N-glycoproteins were localized in the extracellular region, cytoplasm, plasma membrane, and nucleus.

**FIGURE 4:**
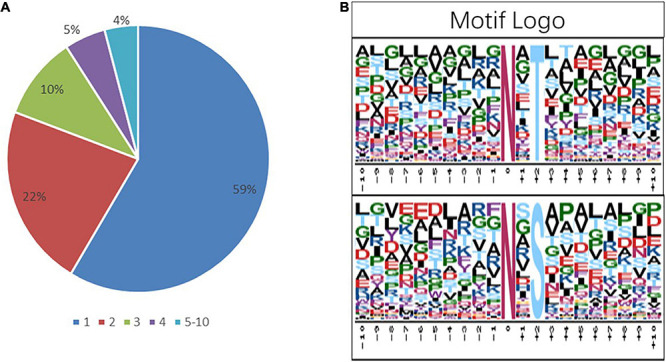
Analysis of the characters of the identified peptides. sequence motifs of the N-glycoproteome data. **(A)** The proportion of the protein containing one or more glycosylation sites in the total glycoprotein; the sector refers to the ratio of each category N-glycoprotein accounting for all N-glycoproteins. **(B)** The distribution of amino acids from −10 position to +10 position of motif N–X–S and motif N–X–T; the central N (at position 0) indicates the N-glycosylation.

To get more insights into the performance of N-glycosylation sites, the N–X–S/T motif was used to examine the occupancy frequency of amino acids at the positions surrounding the specific modification sites (Figure 4 and Supplementary Table 4). In this work, 59% of the identified glycoproteins had only one glycosylation site, and 22, 10, 5, and 4% of the identified glycoproteins had two, three, four, and more glycosylation sites, respectively (Figure 4A). The N-glycosylation sequence motif was also analyzed (Figure 4B). The hydrophobic amino acid (leucine, glycine, and alanine) accounted for the most percentage, except the +4 position, where the glycosylation site is 0 position.

### Functional Analysis of Differentially Expressed Proteins in Proteome

**FIGURE 5:**
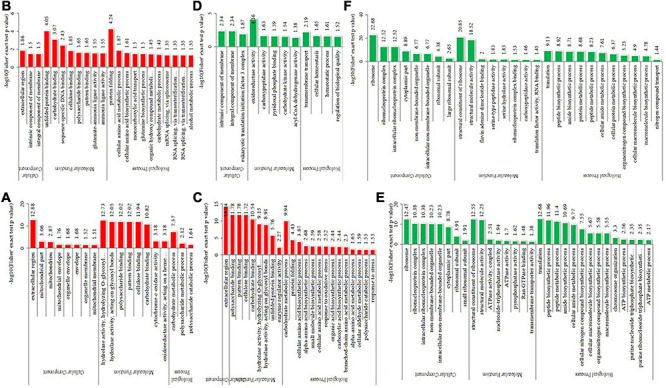
GO-based enrichment analysis of differentially expressed proteins in the different comparisons. **(A–C)** were upregulated categories in T50/T30, T55/T50, and T55/T30, respectively. **(D–F)** were downregulated categories in T50/T30, T55/T50, and T55/T30, respectively.

To detect the significant enrichment tendency of differentially expressed proteins in certain functional types, Gene Ontology (GO) enrichment-based analyses were conducted on proteome data (Figure 5 and Supplementary Table 5). In the category of molecular functions, the high enrichment of many upregulated proteins was realized in carbohydrate or polysaccharide binding and hydrolase activity, pattern binding, and unfolded protein binding. The enrichment of downregulated proteins mostly occurred in the ribosome’s structural constituents, structurally molecular activity, and oxidoreductase activity. For the category of biological processes, the enrichment of upregulated proteins occurred in carbohydrate or polysaccharide metabolic, protein folding, cellular amino acid metabolic or biosynthetic process, and mRNA splicing. The enrichment of downregulated proteins occurred in the translation, biosynthetic or metabolic process of peptide, amide, and protein, and biosynthetic and homeostatic process of cellular macromolecule or nitrogen compound. In the category of cellular components, the high enrichment of upregulated proteins occurred in the extracellular region, membrane or envelope, and mitochondrion. In contrast, the enrichment of downregulated proteins mainly occurred in the ribosome and non-membrane-bound organelle. Therefore, differentially expressed proteins could be highly correlated with carbohydrate metabolism, protein synthesis and metabolism, and protein folding, suggesting that energy metabolism and protein homeostasis play important roles in the thermophily mechanism of *C*. *thermophilum*.

**FIGURE 6:**
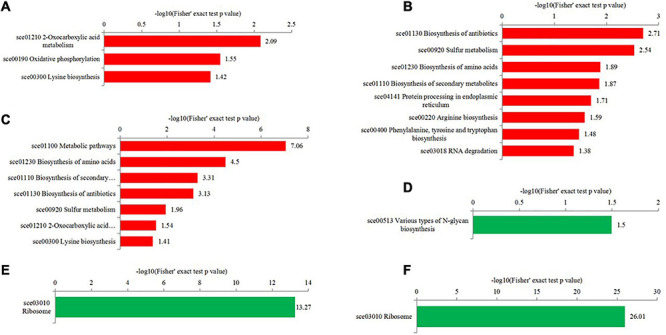
KEGG enrichment analysis of differentially expressed proteins in the different comparisons. **(A–C)** were upregulated categories in T50/T30, T55/T50, and T55/T30, respectively. **(D–F)** were downregulated categories in T50/T30, T55/T50, and T55/T30, respectively.

To get more insights into biological functions, the Kyoto Encyclopedia of Genes and Genomes (KEGG) pathway analyses of differentially expressed proteins were conducted (Figure 6 and Supplementary Table 6). The KEGG pathway is not exactly the same in T50/T30, T55/T50, and T55/T30. In T50/T30, 2-oxocarboxylic acid metabolism, oxidative phosphorylation, and lysine biosynthesis were the upregulated enrichment, whereas varying types of N-glycan biosynthesis were supposed to be the downregulated enrichment. In T55/T50, the biosynthesis of antibiotics, biosynthesis of amino acids, sulfur metabolism, protein processing in the endoplasmic reticulum, biosynthesis of secondary metabolites, and RNA degradation were supposed to be upregulated enrichment. In T55/T30, the biosynthesis of secondary metabolites, metabolic pathways, antibiotics, and amino acids, sulfur metabolism, and 2-oxocarboxylic acid metabolism were considered the upregulated enrichment. In T55/T50 and T55/T30, ribosome was supposed to be the downregulated enrichment.

**FIGURE 7:**
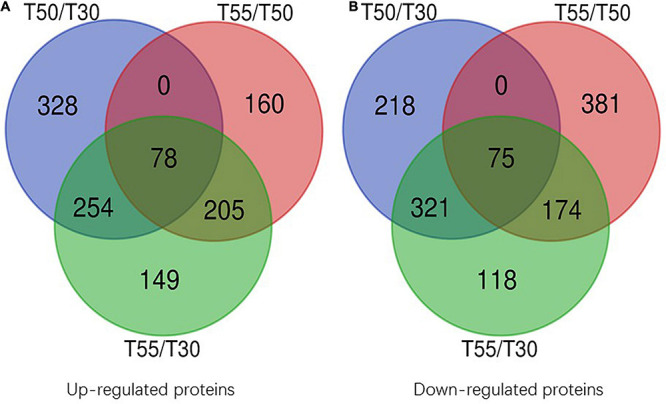
Venn diagram of differentially expressed proteins. **(A)** Venn diagram of up-regulated proteins. **(B)** Venn diagram of down-regulated proteins.

To confirm the commonly and specifically differentially expressed proteins in T50/T30, T55/T50, and T55/T30, Venn diagrams were generated (Figure 7 and Supplementary Table 7). Clearly, 78 upregulated proteins and 75 downregulated proteins were found in T50/T30, T55/T50, and T55/T30. There were 328, 160, and 149 single upregulated proteins and 218, 381, and 118 single downregulated proteins in T50/T30, T55/T50, and T55/T30, respectively. Five proteins with the highest differences in T50/T30, T55/T50, and T55/T30 were list in Table 1.

**TABLE 1:** Five proteins with the highest differences in T50/T30, T55/T50, and T55/T30.

	**Protein accession**	**Protein description**	**Ratio**	**NCBI accession**
T50/T30	G0SGF1	Hypothetical protein	8.739	XP_006696908
	G0S562	RNA helicase-like protein	8.38	XP_006693677
	G0SAF6	Putative heat shock protein	7.906	XP_006694613
	G0SFS0	Dioxygenase-like protein	6.689	XP_006697453
	G0S0C3	Hypothetical protein	6.682	XP_006691475

T55/T50	G0RZQ2	Glutamine synthetase	8.883	XP_006690922
	G0S020	Hypothetical protein	7.182	XP_006691372
	G0SB93	Alcohol dehydrogenase-like protein	5.45	XP_006695295
	G0SGZ0	Putative nitroreductase family protein	5.061	XP_006697097
	G0S7F6	Glutamate dehydrogenase	4.896	XP_006693254

T55/T30	G0S0C3	Hypothetical protein	18.751	XP_006691475
	G0SGZ0	Putative nitroreductase family protein	14.964	XP_006697097
	G0S9J9	Putative cis-trans protein	12.698	XP_006694995
	G0S562	RNA helicase-like protein	12.357	XP_006693677
	G0S020	Hypothetical protein	11.189	XP_006691372

### Functional Analysis of Differentially Expressed N-Glycoproteins in N-Glycoproteome

**FIGURE 8:**
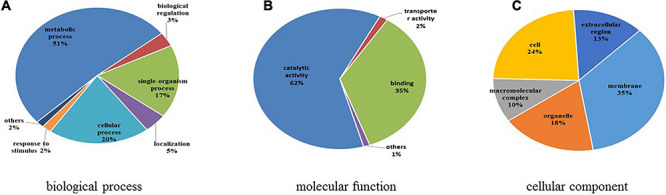
Functional classification of differentially expressed N-glycoproteins. **(A)** Classification based on biological process. **(B)** Classification based on molecular function. **(C)** Classification based on cellular component.

GO functional classification was conducted to illustrate the functions of differentially expressed N-glycoproteins in response to higher temperature in *C*. *thermophilum* (Figure 8 and Supplementary Table 8). Three major protein groups of differentially expressed N-glycoproteins were included in metabolic processes (51%), cellular processes (20%), and single-organism processes (17%). On the basis of the molecular function catalog, most differentially expressed N-glycoproteins were correlated with the catalytic activity (62%) and binding (35%). From cellular component analyses, differentially expressed N-glycoproteins were classified in the membrane (35%), cell (24%), organelle (18%), extracellular region (13%), and macromolecular complex (10%). Therefore, differentially expressed N-glycoproteins were found in various biological processes, such as macromolecule glycosylation, regulation of the cellular biosynthetic process, and organophosphate catabolic process.

**FIGURE 9:**
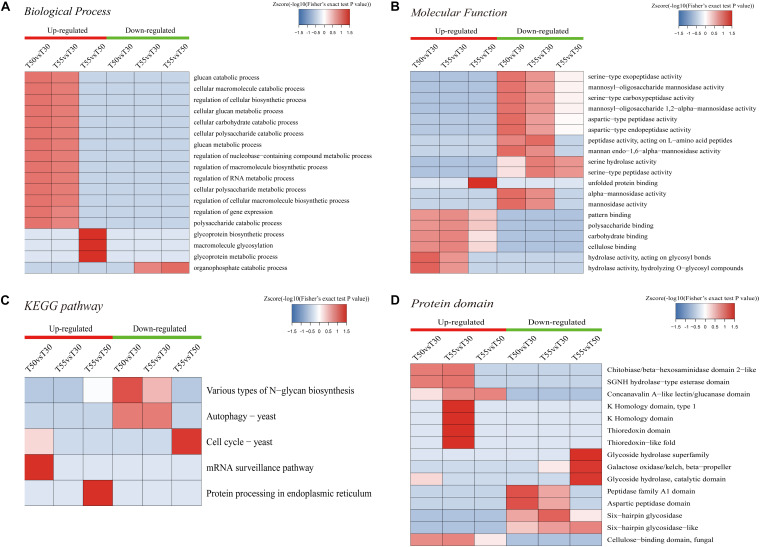
Enrichment-based clustering analysis. **(A)** Biological process. **(B)** Molecular function. **(C)** KEGG pathways. **(D)** Protein domain.

To further illustrate the relevance of differentially expressed N-glycoprotein functions, three kinds of clustering analyses based on enrichment were conducted: GO functional enrichment, protein domain, and KEGG pathway (Figure 9). In GO functional enrichment (Figures 9A–C), the biological process analysis (Figure 9A) showed that upregulated glycoproteins of T55/T50 were mostly enriched in the glycoprotein metabolic macromolecule glycosylation and glycoprotein biosynthetic process. Upregulated glycoproteins of T50/T30 and T55/30 were enriched in the macromolecule biosynthetic and catabolic process, cellular biosynthetic process, nucleobase-containing compound metabolic process, RNA metabolic process, gene expression, carbohydrate catabolic process, and others. Downregulated glycoproteins of T55/T30 and T55/T50 were enriched in the organophosphate catabolic process. In molecular function (Figure 9B), upregulated glycoproteins of T55/T50 were mostly enriched in unfolded protein binding, and upregulated glycoproteins of T50/T30 and T55/T30 were enriched in pattern binding, hydrolase activity, carbohydrate binding, and others. Downregulated glycoproteins were enriched in mannosidase activity and serine/aspartic-type peptidase activity. The KEGG pathway analyses of differentially expressed N-glycoproteins showed five vital pathways in response to temperature changes (Figure 9C). Downregulated glycoproteins of T55/T50 were mostly enriched in the cell cycle, those of T50/T30 were enriched in autophagy and various types of N-glycan biosynthesis, and those of T55/T30 were enriched in autophagy. Upregulated glycoproteins of T50/T30 were enriched in the mRNA surveillance pathway, and those of T55/T50 were enriched in protein processing in the endoplasmic reticulum. To get critically important functional features of proteins, a clustering analysis was then performed using the protein domain (Figure 9D). The upregulated glycoprotein domain was enriched in the K homology domain, thioredoxin domain, SGNH hydrolase-type esterase domain, chitobiase/β-hexosaminidase domain 2-like, concanavalin A-like lectin/glucanase domain, and cellulose-binding domain of fungi. The upregulated glycoprotein domain was enriched in glycoside hydrolase superfamily, galactose oxidase/kelch, β-propeller glycoside hydrolase, catalytic domain, peptidase family A1 domain, six-hairpin glycosidase, and others.

**FIGURE 10:**
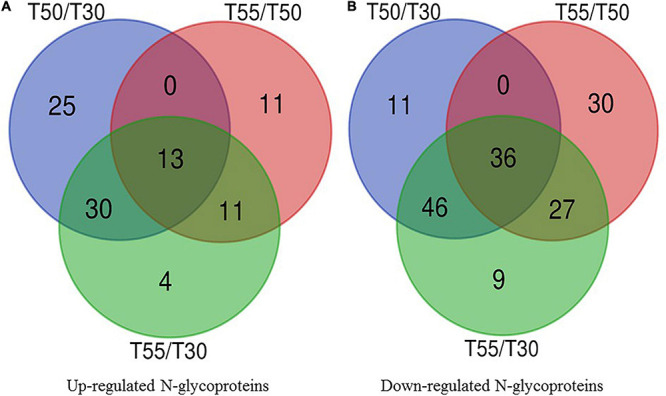
Venn diagram of differentially expressed N-glycoproteins. **(A)** Venn diagram of up-regulated N-glycoproteins. **(B)** Venn diagram of down-regulated N-glycoproteins.

To identify the common and specifically different N-glycoproteins in T50/T30, T55/T50, and T55/T30, Venn diagrams were generated (Figure 10 and Supplementary Table 9). Clearly, 13 upregulated N-glycoproteins and 36 downregulated N-glycoproteins were found in T50/T30, T55/T50, and T55/T30.

### Amino Acid Composition of the Differentially Expressed Proteins

The amino acid composition of differentially expressed proteins was investigated to illustrate the mechanism of *C. thermophilum* thermophily (Table 2). The obvious enrichment of upregulated proteins was found in Glu and Asp residues compared with downregulated proteins in T50/T30. In contrast, the enrichment of upregulated proteins was found in Gly and Pro residues compared with downregulated proteins in T55/T50, suggesting their involvement in high-temperature adaptation (50°C) and high-temperature stress (55°C).

**TABLE 2:** Amino acid composition of the differentially expressed proteins in T50/T30, T55/T50, and T55/T50.

		**A**	**C**	**D**	**E**	**F**	**G**	**H**	**I**	**K**	**L**	**M**	**N**	**P**	**Q**	**R**	**S**	**T**	**V**	**W**	**Y**
T50/T30	UP	9.33 ± 0.08	1.09 ± 0.02	5.78 ± 0.10	7.48 ± 0.11	3.37 ± 0.05	6.52 ± 0.15	2.16 ± 0.01	4.61 ± 0.02	5.51 ± 0.08	8.76 ± 0.14	1.98 ± 0.01	3.49 ± 0.02	6.17 ± 0.09	4.03 ± 0.07	6.22 ± 0.15	7.46 ± 0.04	5.85 ± 0.10	6.21 ± 0.08	1.36 ± 0.05	2.62 ± 0.06
	DOWN	9.2 ± 0.12	1.14 ± 0.02	5.35 ± 0.08	6.34 ± 0.12	3.66 ± 0.01	7.79 ± 0.08	2.27 ± 0.02	4.84 ± 0.03	5.24 ± 0.08	8.46 ± 0.04	2.03 ± 0.02	3.56 ± 0.03	6.54 ± 0.06	3.74 ± 0.07	5.92 ± 0.07	7.1 ± 0.01	5.86 ± 0.04	6.58 ± 0.03	1.5 ± 0.02	2.9 ± 0.02
T55/T50	UP	9.41 ± 0.07	1.17 ± 0.01	5.22 ± 0.02	6.38 ± 0.03	3.45 ± 0.06	7.52 ± 0.03	2.3 ± 0.02	4.65 ± 0.06	5.35 ± 0.14	8.4 ± 0.06	2.05 ± 0.03	3.66 ± 0.01	6.95 ± 0.06	4.18 ± 0.05	5.77 ± 0.05	7.31 ± 0.09	5.86 ± 0.03	6.31 ± 0.05	1.36 ± 0.02	2.71 ± 0.02
	DOWN	9.5 ± 0.04	1.02 ± 0.04	5.69 ± 0.01	7.61 ± 0.09	3.33 ± 0.05	6.49 ± 0.10	2.14 ± 0.02	4.64 ± 0.02	5.83 ± 0.11	8.83 ± 0.05	1.94 ± 0.01	3.46 ± 0.05	5.97 ± 0.06	4.02 ± 0.06	6.4 ± 0.07	7.15 ± 0.08	5.71 ± 0.03	6.28 ± 0.05	1.32 ± 0.04	2.68 ± 0.07
T55/T30	UP	9.32 ± 0.06	1.14 ± 0.01	5.41 ± 0.13	6.91 ± 0.27	3.47 ± 0.02	7.16 ± 0.18	2.24 ± 0.03	4.60 ± 0.03	5.19 ± 0.12	8.36 ± 0.11	1.97 ± 0.01	3.61 ± 0.02	6.86 ± 0.26	4.12 ± 0.03	5.77 ± 0.05	7.45 ± 0.03	6.01 ± 0.05	6.36 ± 0.02	1.41 ± 0.02	2.65 ± 0.06
	DOWN	9.05 ± 0.05	1.13 ± 0.06	5.50 ± 0.18	6.64 ± 0.39	3.57 ± 0.09	7.44 ± 0.42	2.21 ± 0.04	4.81 ± 0.10	5.54 ± 0.26	8.41 ± 0.10	1.96 ± 0.01	3.58 ± 0.06	6.31 ± 0.30	3.71 ± 0.22	6.23 ± 0.27	7.15 ± 0.18	5.85 ± 0.13	6.48 ± 0.13	1.47 ± 0.07	2.93 ± 0.17

**Note: The number refers to the percentage of each amino acid accounting for all the amino acid in up-regulated or down-regulated proteins*.*

## Discussion

The limiting temperature of biological growth depends largely on the thermal stability of the biomolecule, especially the stability of the protein or enzyme. The high-temperature adaptability of proteins has been reported to be related to amino acid distribution and composition (Berka et al., 2011), non-covalent forces (Coquelle et al., 2007), stability of the α-helix (de Souza et al., 2016), solvent-accessible hydrophobic surfaces, and posttranslational modification (Xiao et al., 2008; Zou et al., 2013). A recent study found that cofactor, prosthetic groups, or subunit–subunit interactions help maintain the thermophilic protein’s stability or activity (Brininger et al., 2018).

Although various reports indicated that protein glycosylation has significant effects on protein or enzyme activity and thermal stability (Solá and Griebenow, 2009; Amore et al., 2017; Ge et al., 2018; Kar et al., 2018), cellular glycosylation in response to high temperatures has rarely been reported. In this work, the proteome and N-glycoproteome of *C*. *thermophilum* in response to high temperatures were analyzed by the combination of hydrophilic interaction liquid chromatography (HILIC)-based enrichment and high-resolution liquid chromatography–tandem mass spectroscopy (LC-MS/MS) analyses for the first time. Thus, the widely distributed differentially expressed proteins and N-glycoproteins participated in diversified biological processes.

For functional enrichment analyses of differential proteins, most upregulated proteins were concentrated in carbohydrate metabolic processes, unfolding protein binding, and protein folding. The carbohydrate metabolic process was an important reaction to cell growth and could provide energy for life activities. The carbohydrate metabolic process was associated with the adversity adaptation of heat, cold, and acid (Marceau et al., 2004; Di Cagno et al., 2006; Zhai et al., 2014). Carbohydrate metabolism has been reported to be involved in high-temperature adaptation. Under a high-temperature environment, TCA cycle and oxidative phosphorylation were downregulated in *Mrakia psychrophila* (Su et al., 2016). Under high-temperature stress, carbohydrate and energy metabolism-related proteins were upregulated in *Penicillium marneffei*, *Ustilago maydis*, *Aspergillus niger 3.316*, and *Aspergillus fumigatus* (Chandler et al., 2008; Albrecht et al., 2010; Salmerón-Santiago et al., 2011; Deng et al., 2020). In this study, the enrichment degree of carbohydrate metabolic process-related proteins was positively correlated with the growth of *C*. *thermophilum* in T50/T30 and T55/T30 (Figures 1, 5). In T55/T50, the growth of *C*. *thermophilum* was down, whereas carbohydrate metabolic process-related proteins were still enriched, suggesting that the carbohydrate metabolic process may be relevant to the thermophily mechanism of *C*. *thermophilum.* The correct folding of the protein is critical to its function, and misfolding and aggregation can lead to cell death (Grootjans et al., 2016). The protein misfolding and aggregation caused by high temperature are two of the causes of death of normal-temperature fungi at high temperatures. In a high-temperature environment, the upregulation of protein folding and unfolded protein binding could help the protein fold correctly and degrade misfolded and unfolded proteins (Grootjans et al., 2016). In Figure 5, protein folding and unfolding protein binding were significantly enriched. Therefore, protein folding and unfolding protein binding were supposed to be vital mechanisms in response to the thermophily of *C*. *thermophilum.* In addition, most downregulated proteins were concentrated in the ribosome, biosynthetic and metabolic processes of the peptide, and translation and structural molecule activity. This indicated that high temperature might affect the protein translation of *C*. *thermophilum.*

Protein misfolding is the main feature of heat stress (Jacob et al., 2017). Heat shock proteins have a complex protective mechanism against heat stress, which is important in folding peptide chains and the degradation and removal of denatured proteins to maintain protein homeostasis and cell physiological functions (Voellmy and Boellmann, 2007; Venkatesh and Suzuki, 2017; Morita et al., 2018). As shown in the Venn diagram of differentially expressed proteins (Figure 8), five Hsps G0S4G4, G0SAF6, G0SCQ6, G0SH15, and G0S5V3 were upregulated in T50/T30 and T55/T30, which correspond to XP_006692738, XP_006694613, XP_006696127, XP_006697122, and XP_006693811 in NCBI; four Hsps G0RYB3, G0S8C8, G0S4L1, and G0SBY8 were upregulated in T50/T30, T55/T30, and T55/T50, which correspond to XP_006691141, XP_006692599, XP_006693582, and XP_006695859 in NCBI; and one Hsp G0RYP6 (XP_006691274) was upregulated in T55/T50 and T55/T30, which correspond to XP_006691274 in NCBI. These Hsps contain many types, such as Hsp100, Hsp90, Hsp70, and small Hsp (sHsp) (Radons, 2016). One of the important functions of sHsp is to bind to misfolded proteins to prevent them from gathering (Nakamoto and Vígh, 2007). Recent studies have shown that sHsp could potentially maintain the integrity of membranes under pressure (Sun and MacRae, 2005). Hsp70s has a key role in protein folding, degradation, and disaggregation and is the main player in protein homeostasis (Fernández-Fernández and Valpuesta, 2018; Rosenzweig et al., 2019). Hsp90s play a vital role in protein stability, cell differentiation, and development (Hoter et al., 2018; Doyle et al., 2019). Hsp100s play a decisive role in adapting cells to heat stress. Mutant bacteria and yeast cells lacking active Hsp100 protein are extremely sensitive to high-temperature stress (Mishra and Grover, 2016).

At the +2 position of the N-glycosylation site, the frequency of threonine was 72%, similar to previous reports (Schwarz and Aebi, 2011; Zhang et al., 2016), indicating that the N–X–T motif may have significance in the N-glycosylation process. In the differentially modified GO enrichment-based clustering analysis, unfolded protein binding and glycosylation-related proteins were upregulated enrichment in T55/T50, and carbohydrate metabolic processes are enriched in T50/T30 and T55/T30, indicating that N-glycosylation could affect carbohydrate metabolic processes and protein folding. N-linked glycans could provide blueprints to precisely instruct the folding of protein substrates (Xu and Ng, 2015; Jayaprakash and Surolia, 2017; Macharoen et al., 2020). In KEGG pathway analyses, the mRNA surveillance pathway and protein processing in the endoplasmic reticulum are upregulated enrichment, autophagy, and cell cycle, and varying types of N-glycan biosynthesis were downregulated enrichment. The different kinds of N-glycan biosynthesis were downregulated enrichment in the proteome analysis, suggesting that the thermophily of *C*. *thermophilum* was regulated by multiple glycosylations. N-glycosylation has been reported to affect protein activity and stability (Amore et al., 2017; Ge et al., 2018). Because of the upregulated and downregulated expression of *C*. *thermophilum* N-glycosylated proteins at high temperature, we suggest that both glycosylation and deglycosylation of protein should be a mechanism of the thermophily of *C*. *thermophilum*.

Protein homeostasis is the balance among protein synthesis, transportation, assembly, folding, and degradation, which is important for correct cell function. A variety of strategies were developed by cells to control stress (Fernández-Fernández and Valpuesta, 2018). As shown in GO enrichment analyses, high enrichment of upregulated proteins was found in unfolded protein binding and protein folding (Figure 3), and the enrichment of N-glycosylation-modified different proteins was found in unfolded protein binding and protein processing in the endoplasmic reticulum (Figures 9B,C). On the basis of these data, it was preliminarily speculated that an efficient protein homeostasis pathway is one of the keys for the thermophily of *C*. *thermophilum*, and N-glycosylation may participate in the regulation of protein homeostasis by affecting the functions of related proteins (Tannous et al., 2015; Roth and Zuber, 2017). In a high-temperature environment, the upregulation of unfolded protein binding and protein folding, especially the upregulation of chaperonins and proteasomes (Figure 5), may help the protein fold correctly and degrade misfolded and unfolded proteins (Grootjans et al., 2016). N-glycosylation helps regulate protein homeostasis by changing the function of glycosylation to modify unfolded protein binding and protein processing in endoplasmic reticulum-related proteins.

In this study, Glu and Asp residues were rich in upregulated proteins at the optimum growth temperature of *C*. *thermophilum* (50°C), whereas Gly and Pro residues were rich in upregulated proteins at the maximum growth temperature of *C. thermophilum* (55°C). Proteins from thermophilic fungi had thermostability. Numerous factors affected thermostability, including hydrogen bonds, ion pairs, disulfide bridges, packing, hydrophobic interactions, decreased entropy of unfolding, and intersubunit interactions (Vieille and Zeikus, 2001; Sadeghi et al., 2006; Hait et al., 2020). It was reported that the ratio of charged amino acids (Glu, Arg, Asp, and Lys) was higher in thermophiles and could contribute to increased ion interactions (Trivedi et al., 2006). Gly and Pro residues often occurred in protein turns and affect α-helix stability. Gly was small, had more conformational flexibility, and may contribute to protein thermostability at high-temperature stress (55°C). Proline-rich proteins represent one of the classes of cell wall structural proteins in plants and are involved in different environmental stresses, including high-temperature stress (Cassab, 1998; Priyanka et al., 2010). Pro enhances the stability of the protein by restricting the rotation of the main chain and enhancing the rigidity of the main chain (Watanabe et al., 1994; Kumar and Nussinov, 2001; Feller, 2018). Arg can inhibit protein aggregation, thereby promoting the refolding of inclusion bodies (Tsumoto et al., 2004). The specific recognition of N-glycans can achieve more accurate protein folding (Varki, 2017). Studies have shown that disulfide bonds play an important role in promoting and stabilizing protein folding by reducing the entropy of the unfolded state (Okumura et al., 2011; Arai et al., 2017; Gori et al., 2017; Okada et al., 2019).

## Materials and Methods

### Strains, Culture Media, and Cultivation

The previously isolated *C. thermophilum* CGMCC3.17990 strain was deposited in the China General Microbiological Culture Collection Center (Beijing, China). For harvesting mycelium, *C. thermophilum* was inoculated at 30, 50, and 55°C for 3 days on a CCM medium (Kellner et al., 2016).

### Protein Extraction

First, *C. thermophilum* mycelium was ground by liquid nitrogen, followed by charging the powders into a centrifuge tube (5 mL) and sonicating thrice on ice with an ultrasonic processor (Scientz, Ningbo, China) in a lysis buffer [including 1% Triton X-100, 10 mM dithiothreitol (DTT), 1% protease inhibitor cocktail, 50 μM PR-619, 3 μM TSA, 50 mM NAM, and 2 mM EDTA] (Abu Bakar et al., 2020). Upon adding an equivalent volume of Tris-saturated phenol (pH 8.0), the resulting mixture was vortexed for 5 min. After the centrifugation (4°C, 10 min, 5,000 g) was completed, the phenol in the upper layer was collected in another centrifugation tube. The precipitation of proteins was performed by charging at least four volumes of ammonium sulfate-saturated methanol followed by incubation (−20°C, not < 6 h). After the centrifugation (4°C, 10 min) was completed, and the supernatant was removed, the remaining precipitates were washed with ice-cold methanol once and ice-cold acetone thrice. The re-dissolution of protein was carried out in 8 M urea (Sigma-Aldrich, St. Louis, MO, United States), and the protein concentrations were obtained with a BCA kit (Beyotime Biotechnology, Shanghai, China) based on the manufacturer’s instructions.

### Trypsin Digestion

For digestion, the reduction of protein solution was performed with DTT (5 mM, 56°C, 30 min), and alkylation was performed with iodoacetamide (11 mM, ambient temperature, 15 min, in the dark). The dilution of protein samples was then conducted by adding TEAB (100 mM; Sigma-Aldrich) to <2 M urea. Trypsin (Promega, Madison, WI, United States) was charged at a trypsin-to-protein mass ratio of 1:50 for the first digestion (overnight) and 1:100 for the second digestion (4 h).

### TMT Labeling

After trypsin digestion was completed, the desalting of peptides was conducted with the Strata-X C18 SPE column (Phenomenex, Los Angeles, CA, United States), followed by vacuum drying. The peptides were reconstituted in TEAB (0.5 M) and processed on the basis of the protocol of the manufacturer of the TMT-10plex kit (Thermo Fisher Scientific, Waltham, MA, United States). Generally, the thawing and reconstituting of one unit of TMT reagent were conducted in acetonitrile (ACN). The incubation of peptides was performed at ambient temperature for 2 h, followed by pooling, desalting, and drying during vacuum centrifuging.

### High-Performance Liquid Chromatography (HPLC) Fractionation

The fractionation of tryptic peptides was performed by reversed-phase HPLC (high pH) with the Thermo Betasil C18 column (5-μm particles, 10-mm i.d., 250-mm length; Thermo Fisher Scientific). Generally, the separation of peptides was conducted with ACN at a gradient of 8–32% (pH 9.0) over 1 h to obtain 60 fractions, followed by combining the peptides into four fractions and drying during vacuum centrifugation.

### HILIC Enrichment and Deglycosylation of N-Glycopeptides

Glycopeptide HILIC enrichment was conducted on the basis of previous reports with minor modifications (Zhu et al., 2014; Zhang et al., 2016). For each sample, the re-dissolution of approximately 2 mg of labeled peptides was performed in the enrichment loading buffer (80% ACN/1% trifluoroacetic acid), followed by pipetting into an HILIC tip. After centrifugation (4,000 *g*, 15 min), the HILIC tip was washed with loading buffer (40 μL) thrice. Lastly, the elution of enriched glycopeptides was performed with 50 μL water, followed by lyophilizing to dryness. In the case of the deglycosylation, 200 units of PNGase F in NH_4_HCO_3_ (50 μL, 50 mM) were charged, followed by overnight incubation at 37°C.

### LC-MS/MS Analysis

The dissolution of tryptic peptides was performed in formic acid (0.1%, solvent A), followed by direct loading on a self-made separating column (reverse phase, 15-cm length, 75-μm i.d.). The gradient included an increase of 5–20% solvent B (0.1% formic acid in 98% ACN) within 24 min, an increase of 20–32% within 8 min, and an increase to 80% within 4 min and a holding at 80% for 4 min. All processes were conducted at a fixed flow rate (700 nL/min) on an EASY-nLC 1000 UPLC system (Thermo Fisher Scientific). The peptides were charged to the NSI source, followed by MS/MS in Orbitrap Fusion^TM^ (Thermo Fisher Scientific) mounted to the UPLC online, under an electrospray voltage of 2.0 kV. The *m/z* scan range was 350–1,550 for a full scan, and intact peptides were detected at a resolution of 60,000. The 28 peptides were chosen for MS/MS with NCE setting, and the detection of fragments was conducted at a resolution of 30,000. The variation of the data-dependent process was conducted between one MS scan and 20 MS/MS scans with a dynamic exclusion of 15.0 s. The automatic gain control was set as 5E4, and the constant first mass was 100 *m/z*.

### Database Search

The processing of resultant MS/MS data was performed with the Maxquant searching engine (version 1.5.2.8). The searching for MS/MS against the Proteomes-*C*. *thermophilum*^2^ database concatenated with the reverse decoy database was carried out. Trypsin/P was defined as the cleavage enzyme, which permitted up to two missing cleavages. For precursor ions, the mass tolerance was 20 ppm in the first search and 5 ppm in the main search. For fragment ions, mass tolerance was 0.02 Da.

### Bioinformatics Methods

The GO annotation proteome was collected from the UniProt-GOA database^3^ (Zhang et al., 2016). The subcellular localization prediction software Wolfpsort was employed to forecast subcellular localization. In all protein sequences, the soft motif-x was employed to analyze the model sequences containing amino acids at specific positions of modify-21-mers (10 amino acids upstream and downstream of the site). All sequences in the database were taken as the background, with other parameters as the default. Functional enrichment analyses were conducted on the basis of the GO and KEGG database annotation of differentially expressed proteins. The functional enrichment-based clustering and heatmap were carried out according to previously reported methods (Li et al., 2018).

For protein quantification, the ratios of the TMT reporter ion intensities in MS/MS spectra from raw data sets were used to calculate fold changes between samples. Only peptides unique for a given protein were considered for relative quantitation. For each sample, the quantification was normalized using the median ratio of all the unique peptides. Protein quantitation was calculated from the median ratio of protein corresponding unique peptides. For N-glycosylation site quantification, the ratios of the TMT reporter ion intensities in MS/MS spectra from raw data sets were used to calculate fold changes between samples. For removal of modification caused by changes in protein levels, the ratio of N-glycosylation sites was divided by the ratio of the corresponding protein. Two-tailed Student’s *t*-test was used to examine whether proteins were differentially expressed between samples. Differentially expressed protein enriched pathways were identified by a two-tailed Fisher’s exact test. The pathway with *p*-value <0.05 was considered significant. All calculation and visualization steps were performed in RStudio.

## Conclusion

In this study, the proteome and N-glycoproteome of *C. thermophilum* cultured at different temperatures were analyzed. We identified 3,878 differentially expressed proteins. In addition, we identified 498 glycosylation modification sites among 278 N-glycoproteins. Differentially expressed proteins, which included N-glycoproteins, were associated with large-scale biological processes and metabolic pathways. Our data suggest that protein homeostasis pathways play a key role in the thermophily of *C. thermophilum*. Further, altered patterns of N-glycosylation appear to be correlated with thermophily in this species.

## Data Availability Statement

The datasets presented in this study can be found in https://www.ebi.ac.uk/pride/archive/projects/PXD023311.

## Author Contributions

JG and DL designed the experiments. JG and QL conceived the project, analyzed the data, and wrote the article. DL supervised and complemented the writing. All authors have read and approved the manuscript.

## Conflict of Interest

The authors declare that the research was conducted in the absence of any commercial or financial relationships that could be construed as a potential conflict of interest.

## Footnotes

1 http://www.ebi.ac.uk/pride/

2 http://www.uniprot.org/proteomes/UP000008066

3 http://www.ebi.ac.uk/GOA/

## Supplementary Material

Supplementary Table 1 List of proteins identified and quantified by the TMT analysis in the experiment.

Supplementary Table 2 List of N-glycoproteins identified and quantified by the TMT analysis in the experiment.

Supplementary Table 3 The subcellular location of identified proteins in proteome and N-glycoproteome.

Supplementary Table 4 Amino acid sequence analysis from the −10 to +10 positions around the glycosylated asparagine.

Supplementary Table 5 Enrichment of gene ontology analysis of differentially expressed proteins.

Supplementary Table 6 KEGG pathway of differently expressed proteins.

Supplementary Table 7 Proteins data of venn diagrams in proteome.

Supplementary Table 8 GO annotation of differentially expressed N-glycoproteins.

Supplementary Table 9 Proteins data of venn diagrams in N-glycoproteome.
